# Transcriptomic Analysis of Fuzi Lizhong Decoction for the Treatment of Stomach Ulcers

**DOI:** 10.1155/2020/3291853

**Published:** 2020-02-21

**Authors:** Yang Xin, Haijun Wang, Lei Xu

**Affiliations:** ^1^College of Chemistry and Chemical Engineering, Qiqihar University, Qiqihar 161006, China; ^2^Heilongjiang Provincial Key Laboratory of Catalytic Synthesis for Fine Chemicals, Qiqihar University, Qiqihar 161006, China; ^3^College of Adult and Continuing Education, Qiqihar Medical University, Qiqihar 161006, China

## Abstract

This study aims to understand the treatment of stomach ulcers with FLD and to identify its potential target genes as well as related pathways by transcriptomic analysis. Rat stomach mRNA from a blank group (BG), a model group (MG), an untreated-model group (u-MG), and a treated group (TG) was sequenced. A partial least-squares discriminant analysis (PLS-DA) was used to differentiate the MG from the BG, and the Deseq2 R Package was used to identify differentially expressed genes between these groups. Furthermore, *t*-tests based on transcripts per million (TPM) were used to select different genes between MG and BG and significantly retrieved genes in TG, except for self-retrieved genes in u-MG. Finally, pathways regulated by retrieved genes were analyzed with KEGG database. Results showed that 327 of the 32,623 total detected genes were different (*p* < 0.05) between the MG and BG. Among these genes, eighteen genes were significantly retrieved after rats were treated with FLD in TG, and they were considered as target genes on which FLD acted. In conclusion, FLD was deduced to cure stomach ulcers by affecting glycerolipid metabolism, fatty acid degradation, circadian entrainment, circadian rhythm, and dopaminergic synapse.

## 1. Introduction

Stomach ulcers are common digestive diseases, and they may develop into serious diseases, such as stomach cancer, if not treated in time. In short, stomach ulcer causes the body to be in a condition of poor health. Therefore, it is important to prevent and treat stomach ulcers to maintain health. Therefore, studies on stomach ulcers are of interest to scholars. Öner et al. discovered that some environmental factors involving in lipid peroxidation may disrupt stomach mucosal protection, impair the mucosal barrier, and facilitate the occurrence of stomach ulcers [1]. An increasing number of studies have been committed to explore stomach ulcer pathogenesis [2, 3] and to identify effective pharmaceuticals to treat stomach ulcers [4, 5].

Fuzi Lizhong Decoction (FLD) is derived from “San Yin Ji Bing Zheng Fang Lun” (a traditional Chinese medicine) and consists of equal amounts of *Aconitum carmichaelii* Debeaux, C. Y. Wu, P. H. Raven, and D. Y. Hong, root, salted (ACD); *Zingiber officinale* Roscoe, root, prepared (ZOR); *Glycyrrhiza uralensis* Fisch., root and rhizome, honeyed (GUF); *Atractylodes macrocephala* Koidz., root and rhizome (AMK); and *Codonopsis pilosula* Franch. Nannf., root (CPF). In China, FLD is primarily used to treat digestive disease including chronic gastritis [6], chronic ulcerative colitis [7], and functional dyspepsia [8]. The FLD pill was included in the Chinese Pharmacopoeia (2015 version).

Our previous study had illustrated the curative effect and target metabolites of FLD on stomach ulcers at the metabolic level [9]. Therefore, it is necessary to carry out studies at the molecular level to clarify the mechanism underlying the action of FLD in the treatment of stomach ulcers. Meanwhile, this analysis is expected to identify the same metabolic pathways deduced in our previous study to further examine our previous conclusion. Moreover, it is feasible to study the mechanism of action of FLD on stomach ulcers at the molecular level, according to a previous study on Shenqi Fuzheng's cardioprotective effects [10].

Transcriptomic analysis is a method to study gene expression at the overall RNA level for filtering and identifying risk genes to elucidate mechanisms of disease [11, 12]. Considering the overall concept of Chinese medicine formulas on treating disease and the overall transcriptomic research strategy, transcriptome sequencing of stomach tissue was carried out to investigate the whole effect of FLD on all stomach ulcer-related genes.

The results of transcriptomic analysis usually must be verified by real-time fluorescence quantitative reverse transcription polymerase chain reaction (RT-qPCR), which is an accepted method by scholars [13]. Therefore, this study coupled tissue transcriptome analysis with PCR technology to clarify the mechanism by which Fuzi Lizhong Decoction cures stomach ulcers.

## 2. Experiment

### 2.1. Materials and Methods

#### 2.1.1. Solvent and Medicine

Reserpine (11,17-Dimethoxy-18-[(3,4,5-trimethoxybe-nzoyl)oxy]-yohimban-16-carboxyluic acid methyl ester, lot number: 150908) was provided by Rong He company (Shanghai, China). *ACD* (lot number: 401234024), *ZOR* (lot number: 400072194), *GUF* (lot number: 401135008), *AMK* (lot number: 401004446), and *CPF* (lot number: 401004745) which were produced by Tongrentang Pieces Co., Ltd. (Haozhou, China) were provided by Qi Tai Pharmacy (Qiqihar, China). Total RNA Extractor (TRIzol) was purchased from BBI (Shanghai, China). Qubit RNA HS Assay Kit and Qubit dsDNA HS Assay Kit were supplied by Life Technologies (Massachusetts, USA). HPLC-grade methanol and acetonitrile and analytical-grade ethanol were used. VAHTS™ mRNA-seq V2 Library Prep Kit for Illumina® and VAHTS™ DNA Clean Beads were purchased from Vazyme Co., Ltd. (Nanjing, China). SG Fast qPCR Master Mix (High Rox) (2x) was supplied by BBI (Huntingdon, USA).

#### 2.1.2. Animals and Groups

Twelve male SD (Sprague Dawley) rats (182–228 g) were purchased from Beijing Laboratory Animal Research Center (Beijing, China). Before the experiment, all rats were acclimated to the animal house under humidity of 50 ± 10% and temperature of 23 ± 2°C for seven diurnal cycles. During the seven days, food and water were provided ad libitum. After seven days of acclimation, all rats were randomly separated into four groups with equal number of rats, which was supported by published reference [14]. The whole experimental period consisted of a modeling stage (14 days) and a treatment stage (28 days). During the modeling stage, rats in the blank group (BG) were fed normally, while rats in the model group (MG), treated group (TG), and untreated-model group (u-MG) were fed every other day and intraperitoneally injected with reserpine (0.5 mg kg^−1^) every day, which was a widely used modeling method. During the treatment stage, the rats in TG were administered FLD (1.8 g kg^−1^) intragastrically in accordance with the dosage in “San Yin Ji Bing Zheng Fang Lun,” whereas the untreated-model group was given an equal volume of water.

During the whole experimental period, food intake and body weight were monitored once a week; hair and feces were observed every day; rough and dilute ones served as model characterization.

#### 2.1.3. Sample Preparation

*(1) Medicine Preparation*. A reserpine saline solution was prepared with a concentration of 0.125 mg/ml. FLD was prepared using the reflux extraction method, as follows. First, *ACD*, *CAH*, *GUF*, *AMK*, and *CPF* were weighed 30 g and mixed. Next, the herbal medicine mixtures were soaked in ten volumes of water for 30 minutes and then boiled for 30 minutes/time and 20 minutes/time. Finally, the extract was filtered, mixed, and concentrated to 500 ml. FLD was a multicomponents aqueous solution, which mainly contained alkaloids and phenolic acids compounds, the components in it could be characterised by Ultra Performance Liquid Chromatography-Mass Spectrometry (UPLC-MS) detecting method (see Figures [Supplementary-material supplementary-material-1] and [Supplementary-material supplementary-material-1] in Supplementary [Supplementary-material supplementary-material-1]), and several components named benzoyl aconitine, benzoyl aconitine, atractyl lactone I, atractylenolide II, ginsenoside, emodin, glycyrrhizin, glycyrrhizin, 8-gingerol, and 10-gingerol were quantified as 2.52 *μ*g/mL, 0.11 *μ*g/mL, 0.46 *μ*g/mL, 1.75 *μ*g/mL, 5.8 *μ*g/mL, 0.35 *μ*g/mL, 2.52 *μ*g/mL, 0.98 *μ*g/mL, 6.65 *μ*g/mL, and 2.71 *μ*g/mL based on our preliminary study.

*(2) Extraction of Total RNA*. Stomach tissue samples from twelve rats were collected immediately at the end of the modeling stage and the treatment stage. Tissue samples were homogenized with total RNA Extractor (TRIzol) and then allowed to rest for 10 min to separate nucleic acids from nuclear proteins. Then 0.2 mL of chloroform was added, and the mixture was vortexed for 15 s. After resting for 3 min at room temperature, the mixture was centrifuged at 12000 ×*g* at 4°C for 10 min. Further, the supernatant was removed and mixed with an equal volume isopropanol before centrifugation at 12000 rpm at 4°C for 10 min. The precipitate was then washed with 75% ethanol followed by a 3 min centrifugation at 12000 ×*g* at 4°C. The sample was then dried at room temperature for 10 min, and the RNA was dissolved with 50 *μ*L of RNase-free ddH_2_O. The total RNA quality was inspected by agarose gel electrophoresis.

#### 2.1.4. Library Construction

*(1) Separation of mRNA from Qualified Total RNA*. First, the mRNA Capture Beads were removed from 2 to 8°C and placed at room temperature. Second, 1 *μ*g of total RNA was dissolved in 50 *μ*L of nuclease-free water in a nuclease-free tube and placed on ice for use. Third, 50 *μ*L of mRNA Capture Beads was mixed in the total RNA. The sample was then placed in a PCR machine at 65°C for 5 min for RNA denaturation. After incubating at room temperature for 5 min, the supernatant was removed after the mixture was on a magnetic rack for 5 min. The down layer was mixed with 200 *μ*L of Bead Wash Buffer, and the supernatant was removed after the mixture rested on a magnetic rack for 5 min. The sample was removed from the magnetic stand, and the magnetic beads were resuspended in 50 *μ*L of Tris Buffer and mixed thoroughly. The samples were placed in the PCR machine, held at 80°C for 2 min and at 25°C, and then the mRNA was eluted. Next, 50 *μ*L of Bead Binding Buffer was added to the tube and pipetted 6 times to mix thoroughly. The tube was placed at room temperature for 5 min to make the mRNA bind to the magnetic beads. The samples were placed on the magnetic stand for 5 min to separate the mRNA from total RNA, and then the supernatant was removed carefully. The samples were removed from the magnetic stand, rinsed with 200 *μ*L of Bead Wash Buffer 6 times, and mixed thoroughly, and then they were incubated on the magnetic stand for 5 min before the supernatant was aspirated. The samples were removed from the magnetic stand, and the magnetic beads were resuspended with 19.5 *μ*L of Frag/Prime Buffer before being mixed 6 times by pipetting. The samples were placed in the PCR machine at 94°C for 5 min and held at 4°C. The samples were placed on the magnetic stand for 5 min, and then 17 *μ*L of the supernatant was transferred to a new nuclease-free centrifuge tube and immediately used in the first-strand synthesis reaction.

#### 2.1.5. Synthesis of Double-Stranded cDNA

The 1st Strand Buffer was taken out from −20°C and thawed to prepare reaction solution for the first-strand cDNA synthesis:

  Fragmented mRNA-17 *μ*L
  1st Strand Buffer-6 *μ*L
  1st Strand Enzyme Mix-2 *μ*L

After incubating the mixture at 25°C for 10 min, 42°C for 15 min, and 70°C for 15 min, the second-strand synthesis reaction was immediately carried out.

The 2nd Strand Buffer was taken out from −20°C and thawed to prepare reaction solution for the second-strand cDNA synthesis:

  1st Strand cDNA-25 *μ*L
  2nd Strand Buffer-20 *μ*L
  2nd Strand Enzyme Mix-5 *μ*L

The reaction was carried out at 16°C for 60 min. First, 90 *μ*L (1.8x) of VAHTSTM DNA Clean Beads was added to purify the double-stranded cDNA. Next, 62.5 *μ*L of nuclease-free water was added to dissolve the beads, and 60 *μ*L of the supernatant was carefully pipetted into a new nuclease-free centrifuge tube.

The End Prep Mix was removed from −20°C and thawed to prepare the reaction solution for the end-repair reaction solution:

  ds cDNA-60 *μ*L
  End Prep Mix-40 *μ*L

The reaction was carried out at 30°C for 30 min. First, 160 *μ*L (1.6x) of VAHTSTM DNA Clean Bead was added to purify the end-repaired product. Next, 20 *μ*L of nuclease-free water was added to dissolve the beads, and 17.5 *μ*L of the supernatant was carefully pipetted into a new nuclease-free centrifuge tube.

*(1) End dA-Tailing*. The dA-Tailing Buffer Mix was removed from −20°C and thawed to prepare the terminal dA-Tailing reaction solution:

  The purified end-repaired product-17.5 *μ*L
  dA-Tailing Buffer Mix-10 *μ*L
  dA-Tailing Enzyme Mix-2.5 *μ*L

After incubating the solution at 37°C for 30 min and 70°C for 5 min, the linker reaction was carried out immediately.

*(2) Connector Connection*. The RNA Adapter was removed from −20°C and thawed to prepare a ligation reaction solution:

  dA-Tailing production-30 *μ*L
  Ligation Mix-2.5 *μ*L
  RNA Adapter (with barcode, 1 *μ*M)-2.5 *μ*L

The reaction was carried out at 30°C for 10 min and stopped with 5 *μ*L of Stop Ligation Mix.

#### 2.1.6. Ligation Product Purification and Fragment Size Sorting

First, 40 *μ*L of (1x) VAHTSTM DNA Clean Beads was added to purify the ligation product, and 102.5 *μ*L of nuclease-free water was added to dissolve the beads. Next, 100 *μ*L of the supernatant was transferred into a new nuclease-free centrifuge tube.

To purify the ligation product, 70 *μ*L of (0.7x) VAHTSTM DNA Clean Beads was added, and 155 *μ*L of the supernatant was pipetted into a new nuclease-free centrifuge tube. Next, 10 *μ*L of (0.1x) VAHTSTM DNA Clean Beads was added to purify the ligation product, 22.5 *μ*L of nuclease-free water was added to dissolve the beads, and 20 *μ*L of the supernatant was pipetted into a new nuclease-free centrifuge tube.

#### 2.1.7. Library Amplification

The PCR Primer Mix and Amplification Mix 1 were removed from −20°C, thawed, and mixed to prepare a PCR solution:

  Purified linker product-20 *μ*L
  PCR Primer Mix-5 *μ*L
  Amplification Mix-1–25 *μ*L

*(1)PCR Reaction Condition:*

  98°C, 30 s;
  98°C, 10 s;
  60°C, 30 s; 15 cycles
  72°C, 30 s;
  72°C, 5 min;
  4°C, ∞.

To purify the ligation product, 50 *μ*L of (1x) VAHTSTM DNA Clean Beads was added, and 25 *μ*L of nuclease-free water was added to dissolve the beads. Next, 22.5 *μ*L of the supernatant was pipetted into a new nuclease-free centrifuge tube.

#### 2.1.8. Quantitative Mixing

The recovered DNA was accurately quantified using the Qubit 2.0 DNA Assay Kit for sequencing in a 1 : 1 ratio.

#### 2.1.9. Transcriptome Sequencing

Transcriptome sequencing was carried out on the Illumina HiSeq platform (Illumina Co., HiSeq 2500, USA).

#### 2.1.10. Real-Time Fluorescent Quantitative Reverse Transcriptase Polymerase Chain Reaction (RT-qPCR)

The relative quantification of genes from BG (three biological replications), MG (three biological replications), and TG (three biological replications) was carried out on an ABI StepOnePlus PCR instrument (ABI, USA). The reaction mixture was prepared as listed in Table 1, and the PCR conditions are shown in Table 2.

#### 2.1.11. Preparation of Stomach Tissue for Pathological Images

All stomach tissue samples were prepared for pathological sectioning, which were sequentially dehydrated, infiltrated with wax, embedded, imaged, and stained.

### 2.2. Sample Detection

The concentration of the total RNA was measured with a Qubit 2.0 fluorometer (Invitrogen Co., Q32866, USA), and its quality was assessed by agarose gel electrophoresis (Liu Yi Co., DYY-11, China). Electrophoresis images were acquired by a bioelectrophoresis image analysis system (Furi Co., FR-980A, China). The images of stomach pathologies were acquired on a NIKON CI-S microscope (Tokyo, Japan).

### 2.3. Data Analysis with the DS-FI2 Imaging System

#### 2.3.1. Alignment Analysis of Reference Sequence

Qualitative sequences were aligned to the reference genome using HISAT2 software (version 2.1.0), and the results were compiled by the RSeQC software (version 2.6.1). Normal distribution of the duplicate reads was ascertained through RSeQC.

#### 2.3.2. Analysis of Transcriptome Sequence

The quality of raw sequencing data was assessed via FastQC (version 0.11.2) with GO.db (version 3.3.0). Mass shearing was conducted by Trimmomatic (version 0.36) of vegan (version 2.0-10) for acquiring accurate data. Samples of valid data were compared to a reference genome, and mapping information was compiled with HISAT2 (2.1.0). Based on the comparison results, the duplicate reads and the distribution of inserted passages were analyzed by RSeQC (2.6.1). Homogeneity distribution and genomic structure distribution were analyzed by Qualimap (2.2.1). Analysis of gene coverage ratio and sequence distribution on chromosomes was conducted by BEDTools (2.26.0). BCFtools (version 1.5) was used to find possible SNP loci based on the mapping results. The effects of SNP loci on genes were determined by SnpEff (version 2.36). Then sequences were mapped onto the assembled genome using StringTie (1.3.3b) and were compared to known gene models to reveal new transcribed regions with GffCompare (0.10.1). Then, variable shear analysis and fusion gene analysis were conducted with ASprofier (version 1.0.4) and EricScript (version 0.55), respectively.

#### 2.3.3. Analysis of Gene Expression

Partial least-squares discriminant analysis (PLS-DA) which is a valuable visualization tool for showing the different groups separated in the dimensional space was conducted by *R* package (version 3.0.2). It was used to observe the overall classification of different groups from macro perspective; farther distance between groups will indicate more difference. Gene expression levels were evaluated through TPM (transcripts per million) as calculated by StringTie (1.3.3b) software.

#### 2.3.4. Statistical Analysis of Gene Expression Difference

Analysis for expression differences between the BG and MG was performed by using DESeq2 (1.12.4), both mean TPM value and ∣ log_2_FoldChange ∣ serverd as cut-offs for screening out differentially expressed genes. Further, one-way analysis of variance (ANOVA) after variance homogeneity test was applied with the help of IBM SPSS Statistics 19 software for picking up significantly different genes (*p* < 0.05 was considered as statistically significant) as follows. Firstly, ANOVA of genes TPM value between BG and MG was conducted for picking up stomach ulcer-related genes. Secondly, ANOVA of genes TPM value between MG and TG was conducted for selecting significantly retrieved genes. Thirdly, ANOVA of genes TPM between MG and u-MG was using for selecting self-retrieved genes. Lastly, target genes of FLD were initially identified based on significantly retrieved genes except for self-retrieved ones.

In the end, ∆Ct value from RT-qPCR data for BG, MG, and TG was calculated and AONVA of ∆Ct among three groups was conducted to ascertain target genes, and *p* < 0.05 was considered as statistically significant.

### 2.4. Ethics Statement

This study was approved by the Beijing Laboratory Animal Research Center (SYXK (Beijing) 2015–0046) and complied with all national and international guidelines for research involving animals. After the experiments, all rats were euthanized with CO_2_ under ether anesthesia to minimize suffering.

## 3. Results

### 3.1. Total RNA Quality Inspection

The result of the total RNA quality inspection was shown in Table 3. The RIN values of all RNA samples were above 7.0, which met the requirements for second-generation sequencing library construction.

### 3.2. Statistics of Gene Expression

Analysis of transcriptome sequence showed that there were a total of 21,170 new predicted transcripts. Gene expression can be reflected through the abundance of the corresponding transcript. Higher transcript abundance correlates with higher gene expression. In this study, the gene expression level was estimated by the number of sequencing reads that were located in the genomic region or exonic regions. Because the reads count can be affected by gene length and sequencing depth, their absolute quantity may not be proportional to the real expression level. Therefore, the TPM (transcripts per million) was calculated by StringTie (1.3.3b) software to correct the above effect and evaluate the gene expression level. There were a total of 32,623 genes with calculated expression profiles.

### 3.3. Replication Examination

Biological replication is essential for biological experiments, which could prove that the biological experiment was reproducible and ensure the accuracy of the subsequent differentially genetic analysis. In this study, Kendall and Spearman were calculated as correlation coefficient for evaluating reliability of results. Kendall results between samples of BG were 0.8502, 0.8764, and 0.8282 and those of MG were 0.7657, 0.8433, and 0.8333, respectively. Spearman results between samples of BG were 0.9369, 0.9554, and 0.9274 and those of MG were 0.8957, 0.9419, and 0.9319, respectively. The closer to 1 the correlation coefficient becomes, the higher similarity of expression patterns between samples is.

### 3.4. Stomach Ulcer-Related Genes

**Figure 1:**
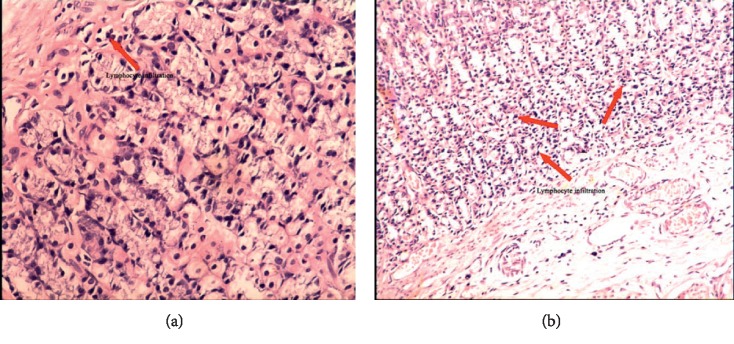
Pathological photos of blank rat (a ×20) and model rat (b ×20). (a) Pathological photos of blank rat (×20). (b). Pathological photos of model rat (×20).

**Figure 2:**
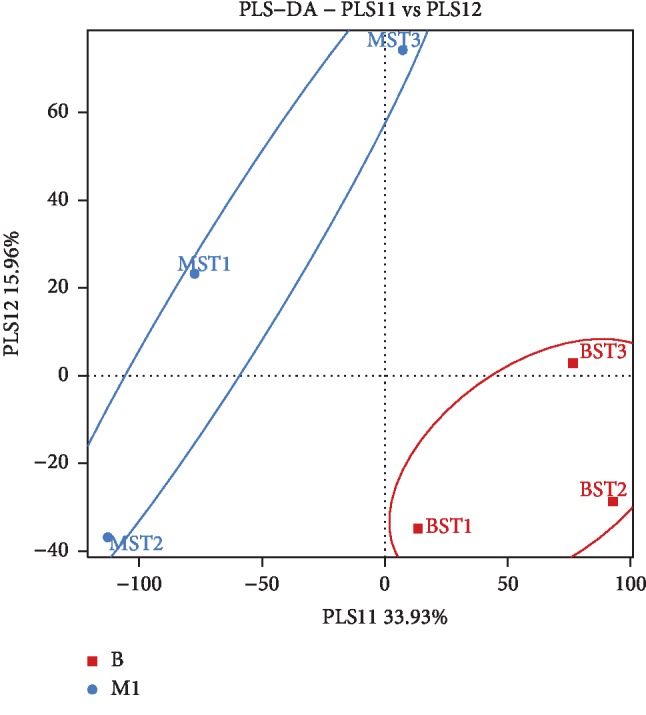
PLS-DA score plot from different groups during modeling period (■: blank group, ●: model group).

Stomach ulcers could be judged through three aspects in this paper, including apparent behavior, pathology, and pattern recognition. In the model group, abnormal behaviors, such as reduced food intake and body weight, rough hair, and diarrhea, were observed. Moreover, increased lymphocyte infiltration in the stomach tissue of model rats was observed in the whole pathological sections. An enlarged image of stomach pathological sections was shown in Figure 1, and visible increased lymphocytes can be seen in the model group compared with the blank group. Meanwhile, stomach damage in each rat was scored based on the degree of lymphocyte infiltration in the whole pathological slice; pathological score showed that the average damage scores of the BG and MG were 0.00 and 2.00 ± 0.4. The higher the scores, the more serious the ulcer. Difference between BG and MG showed statistical significance (*p* < 0.05; see Table 4) based on ANOVA. The pattern recognition of PLS-DA score plot (Figure 2) demonstrates the classification of different groups. In Figure 2, each point represents an individual sample; the same color point represents the same group; it can be seen that the model group is well separated from the blank group. This result indicated that there were endogenous changes in MG versus BG. All the above findings indicated that the stomach ulcer modeling succeeded.

**Figure 3:**
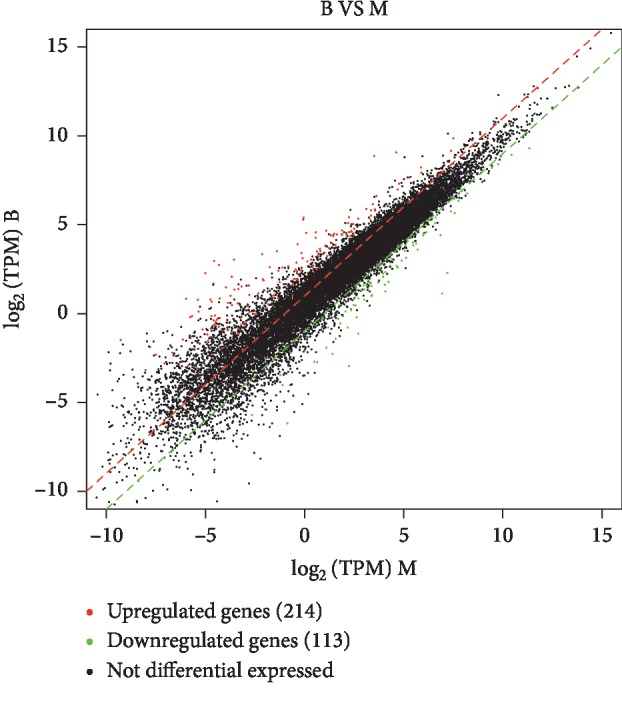
Scatter plots of different genes between blank group and model group.

**Figure 4:**
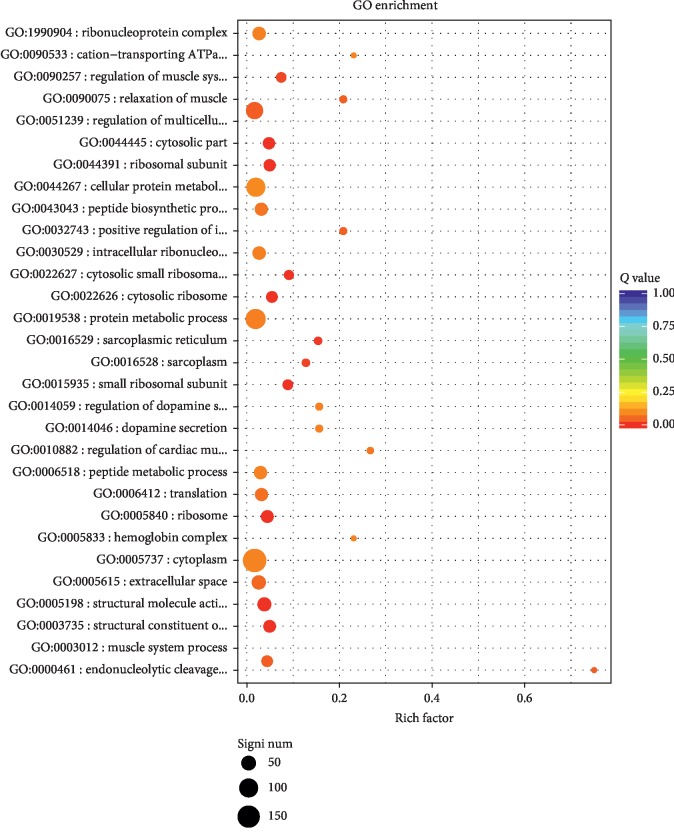
Function enrichment scatter of differential genes between blank group and model group.

To identify stomach ulcer-related genes, gene expression differences between the BG and MG were analyzed through DESeq2. Mean TPM value > 0.1 in the blank group and ∣ log_2_FoldChange ∣  > 1 served as cut-offs for screening out differentially expressed genes among 32,623 detected genes. A total of 327 (214 upregulated and 113 downregulated) genes were identified and colored in Figure 3, which were considered as stomach ulcer-related genes. ANOVA was applied for evaluating the significant difference of different genes between BG and MG. Information of top 20 significantly up and down genes was listed in Table 5. GO (gene ontology) enrichment analysis of differential genes was carried out by the *R* igraph package (1.0.1), and the top 30 most significant functions were shown in Figure 4. In Figure 4, the point size represents the number of genes with the same function; the bigger the point size, the more the different genes. The color of the dots represents the significance of the function, the most significant with red and the least significant with blue. The rich factor represents the top differential genes among the total detected genes annotated with the same function. Results showed that stomach ulcer affected numerous biological functions involving structural molecule activity, cellular component, and biological process. Among top 30 GO, structural constituent of ribosome and structural molecule activity belonged to molecular function, small ribosomal subunit, ribosome, ribosomal subunit, cytosolic ribosome, cytosolic part, cytosolic small ribosomal subunit, sarcoplasmic reticulum, sarcoplasm, extracellular space, intracellular ribonucleoprotein complex, ribonucleoprotein complex, cytoplasm, hemoglobin complex, and cation-transporting ATPase complex, cellular protein metabolic process belonged to cellular component, regulation of muscle system process, regulation of multicellular organismal process, muscle system process, endonucleolytic cleavage to generate mature 3′-end of SSU-rRNA from (SSU-rRNA, 5.8S rRNA, and LSU-rRNA), relaxation of muscle, translation, peptide biosynthetic process, regulation of cardiac muscle contraction by calcium ion signaling, protein metabolic process, dopamine secretion, and regulation of dopamine secretion, and peptide metabolic process belonged to biological process.

### 3.5. Curative Effect of FLD

**Figure 5:**
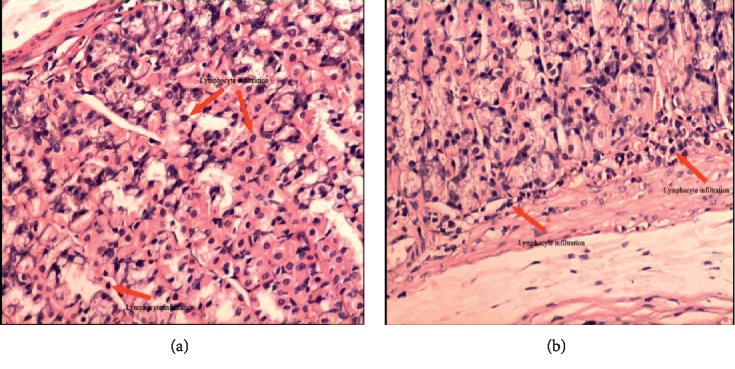
Pathological photos of untreated-model rat (a, ×20) and treated rat (b, ×20).

**Figure 6:**
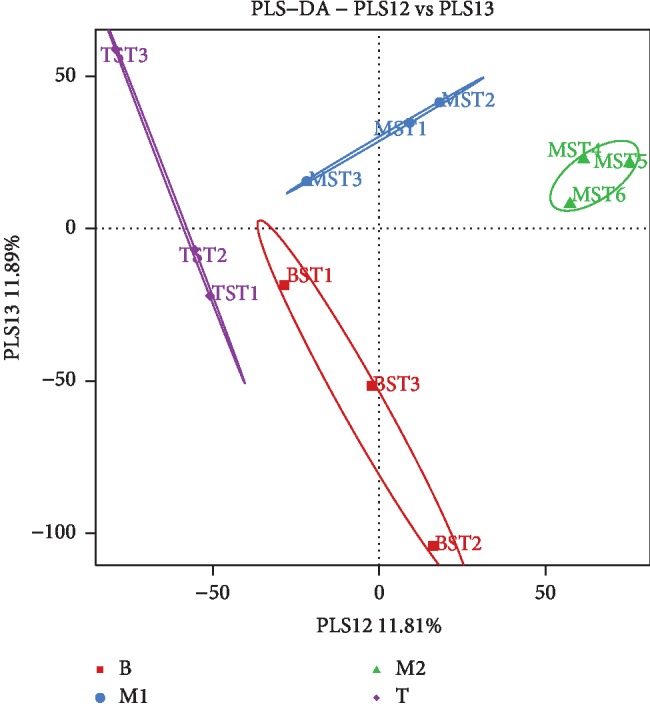
: PLS-DA score plot from different groups during modeling period (■: blank group, ●: model group, ▲: untreated-model group, and ◆: treated-model group).

The curative effect of FLD on stomach ulcers was evaluated on the aspects of apparent behavior, stomach pathology, and PLS-DA of RNA sequencing from stomach tissue. After treatment, the apparent behavior and stomach tissue histology improved nearly to be normal. An enlarged image of the stomach histological section is shown in Figure 5. Fewer lymphocytes were observed in the treated group compared with the untreated-model group, which indicated that the retrieve of stomach tissue was due to the intervention of FLD. Meanwhile, pathological score showed that the average damage scores of the u-MG and TG were 2.67 ± 0.4 and 1.00 ± 0.00. The lower the scores, the better the stomach retrieve. Scores of lymphocyte infiltration and angiogenesis in u-MG compared with those in BG showed statistical significance (*p* < 0.05; see Table 4) which indicated that the extent of damage was not mitigated in u-MG in which the rats were not treated with FLD. Scores in TG were near BG, which could demonstrate the curative effect of FLD, although there was no significant difference between them. In addition, the TG was more similar to the BG rather than the MG and u-MG in Figure 6. The above results indicated that FLD was effective in treating stomach ulcers.

### 3.6. Target Genes of FLD

**Figure 7:**
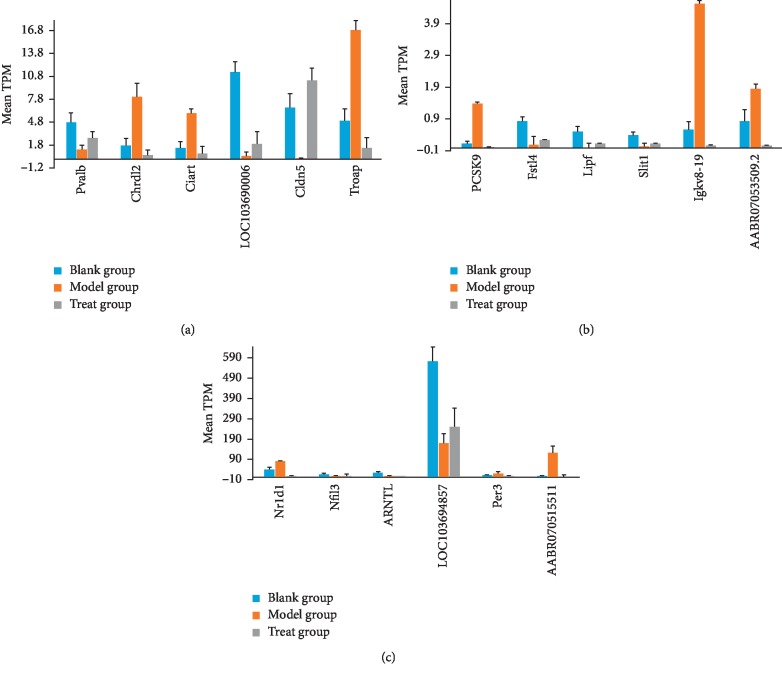
Histogram of mean TPM value of significant retrieved genes in different group (mean + SD).

The identification of target genes of FLD was the final goal of this study. Based on the value of mean TPM, 105 of 327 differentially expressed genes showed callback in the treated-model group on different degrees. Eighteen of them including Pcsk9, Pvalb, Fstl4, Nr1d1, Nfil3, Arntl, Chrdl2, Per3, Lipf, Slit1, Ciart, LOC103690006, Cldn5, Igkv8-19, AABR07051551.1, AABR07053509.2, Troap, and LOC103694857 were significantly different in MG versus BG and TG versus MG but insignificantly different in TG versus BG (Figure 7) and were considered as target genes upon which FLD acts.

### 3.7. Real-Time Fluorescent Quantitative Reverse Transcriptase Polymerase Chain Reaction (RT-qPCR)

**Figure 8:**
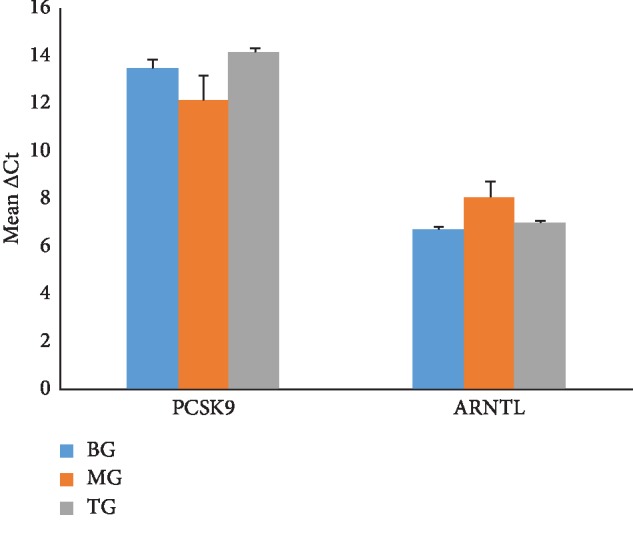
Histogram of mean ∆Ct value of Pcsk9 and Arntl in different group (mean + SEM).

Further, among the 18 potential target genes described above, Pcsk9 and Arnt1 were quantified by RT-qPCR. The average cycle threshold (Ct) of target genes and reference gene glyceraldehyde-3-phosphate dehydrogenase (gapdh) in each group was shown in Table 6 (corresponding bar graph was shown in Figure 8). In order to compare expression levels of target gene in different group, ∆Ct, ∆∆Ct, and 2^−(∆∆Ct)^ were calculated; meanwhile, ANOVA was carried out between MG (or TG) and BG based on ∆Ct; significant difference between groups was demonstrated with *p* < 0.05. In Table 6, ∆Ct represented the difference between the target gene and gapdh in the same group; ∆∆Ct was the difference of ∆Ct between MG (or TG) and BG. Lower ∆Ct values correlated with higher gene expression. Results of Pcsk9 and Arntl showed that there were significant difference between MG and BG and insignificant difference between TG and BG, which were consistent with the findings of the transcriptomic analysis. Although other genes need further testing, the current results could powerfully demonstrate the value of transcriptome sequencing in clarifying the efficacy of Chinese medicine.

## 4. Discussion

This study attempted to clarify the curative effect of Chinese medicine formula “FLD” on stomach ulcers as well as screen out target genes on which it acted. At present, strategy of metabolomics is a common application for deducing genes on which Chinese medicine is acting [15]. Nevertheless, it could only discover genes which regulated endogenous metabolites involved in metabolic pathways; namely, not all target genes could be found out. So, high-throughput sequencing was applied in this study for screening out differential genes and a total of 327 genes were identified. Meanwhile, gene ontology enrichment was carried out for revealing stomach ulcer-related biological functions, and numerous GO were discovered, while only top 30 GO were concerned in this study. Results of GO enrichment demonstrated the related biological function with stomach ulcer such as relaxation of muscle and dopamine secretion [16].

For clarifying the target genes of FLD, ANOVA of gene expression level in different groups was carried out and eighteen ones were deduced. Among these eighteen genes, although not all of them were reported to be related to stomach ulcer, several had been shown to be involved directly or indirectly as follows.

Pcsk9, which was a member of the subtilisin protease family, had been reported as a new target in dyslipidemia treatment [17]. Pcsk9 had a diurnal rhythm synchronous with cholesterol synthesis, and fasting might reduce plasma pcsk9 levels in humans [18, 19]. Moderate alterations were observed in the levels of cholesterol in rats with aspirin-induced ulcers [20]. Our previous study also showed disturbed phospholipid metabolism in rats with stomach ulcers. Therefore, abnormal expression of Pcsk9 in model rats in this study is explainable. Based on the above findings, FLD might treat stomach ulcers by regulating phospholipid metabolism via the pcsk9 gene.

Nrldl was a gene that participates in the circadian rhythm pathway, and the circadian rhythm in some gastric protective factors may render the gastric mucosa vulnerable to injury in a circadian fashion [21]. Therefore, Nrldl might contribute to gastric mucosa protection or destruction.

This study revealed that Arntl was downregulated in model rats, which suggested that the dopaminergic synapse pathway was affected when stomach ulcers occurred. Dopamine was a neurotransmitter secreted by the brain, and its enhanced synthesis was linked with the development of gastric ulcers [22]. The secretion of dopamine increased in rats with stomach ulcers and the expression of Arntl would decrease.

Per3, which was one of the final products of L-glutamate in the circadian entrainment pathway, was found to be upregulated in the model group in the present study. Therefore, it was deduced that the levels of L-glutamate and NO increased. A previous study indicated that acetic acid-induced abdominal writhing was associated with NO production and the glutamatergic system [23]. Writhing may happen when ulcers cause abdominal pain, and glutamate was the major excitatory neurotransmitter involved in nociceptive signal transmission. Therefore, per3 might be one of the regulatory genes associated with gastric ulcers.

Large stomach ulcers might penetrate the pancreas [24], so Lipf secreted from the pancreas in organisms with stomach ulcers would be expressed abnormally. Lipf was one of the pivotal genes in the glycerolipid metabolism pathway, and it regulated fat digestion and absorption. It was downregulated in the model group compared with the blank group, implying that glycerolipid metabolism was disturbed when stomach ulcers occurred.

**Figure 9:**
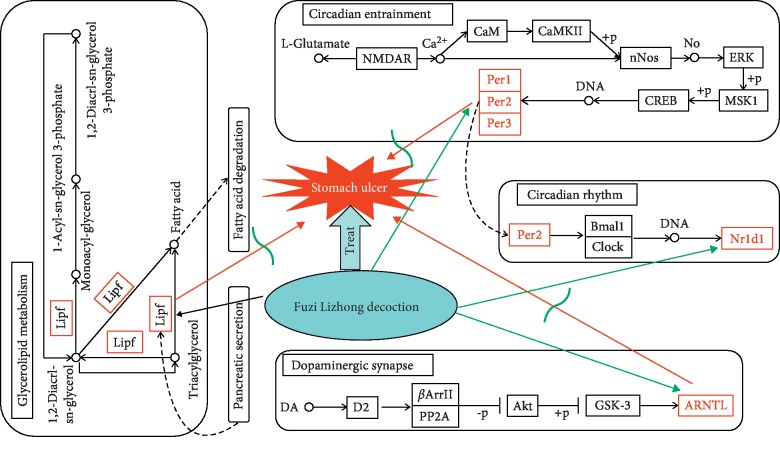
Deduced metabolic pathways of FLD treating stomach ulcer.

Based on the above results, it could be deduced that FLD cured stomach ulcers by affecting glycerolipid metabolism, fatty acid degradation, circadian entrainment, circadian rhythm, and dopaminergic synapses. The deduced functional network based on this study was shown in Figure 9.

## 5. Conclusion

Based on our results, the curative effect and mechanism of FLD on stomach ulcers were assessed by tissue transcriptome analysis coupled with PCR technology. Results indicated that FLD could be used to treat stomach ulcers, and its curative effect was preferable. By reference sequence alignment analysis, the total new genes were predicted. The expression of 32,623 genes was analyzed. *t*-tests were applied to identify stomach ulcer-related genes, resulting in the identification of 327 genes with *p* < 0.05. Eighteen differentially expressed genes showed significant callback in the treated group based on the mean TMP value and were confirmed as target genes of FLD as well. These results indicated that the pharmacological effect of FLD on stomach ulcers reflected its impact on glycerolipid metabolism, fatty acid degradation, circadian entrainment, circadian rhythm, and dopaminergic synapses. FLD was expected to be developed as a beneficial pharmaceutical for the stomach in the future.

## Figures and Tables

**Table 1:** Preparation of the reaction mixture.

Reaction component	Concentration	Volume (*μ*L)
SYPR Green qPCR master mix	2x	10
Primer F (10 *μ*M)	10 *μ*M	0.4
Primer *R* (10 *μ*M)	10 *μ*M	0.4
ddH_2_0		7.2
Template (cDNA)		2

**Table 2:** PCR condition.

Thermal cycler	Initial steps	Times and temperatures		
		Each of 45 cycles		
		Melt	Annealed	Extended
PCR	HOLD	CYCLE		
	3 min 95°C	7 s 95°C	10 s 57°C	15 s 72°C

**Table 3:** Result of total RNA quality inspection.

Sample name	Sample type	RNA concentration (ng/*μ*L)	RIN	Qualified or not
Blank stomach tissue 1 (BST1)	RNA	492	8.3	Yes
Blank stomach tissue 2 (BST2)	RNA	500	8.3	Yes
Blank stomach tissue 3 (BST3)	RNA	430	8.0	Yes
Model stomach tissue 1 (MST1)	RNA	580	9.0	Yes
Model stomach tissue 2 (MST2)	RNA	340	8.0	Yes
Model stomach tissue 3 (MST3)	RNA	242	7.7	Yes
Untreated-model stomach tissue 1 (MST4)	RNA	440	8.0	Yes
Untreated-model stomach tissue 2 (MST5)	RNA	500	9.0	Yes
Untreated-model stomach tissue 3 (MST6)	RNA	528	8.0	Yes
Treated stomach tissue 1 (TST1)	RNA	480	8.7	Yes
Treated stomach tissue 2 (TST2)	RNA	520	9.0	Yes
Treated stomach tissue 3 (TST3)	RNA	506	8.8	Yes

**Table 4:** Pathological score of stomach tissue in different groups.

Group name	Sample name	Lymphocyte infiltration (0–3)	*p* value	Angiogenesis (0–3)	*p* value
BG	Blank stomach tissue 1	0	—	0	—
	Blank stomach tissue 2	0		0	
	Blank stomach tissue 3	0		0	
	Blank stomach tissue 4	0		0	
	Blank stomach tissue 5	0		0	

MG	Model stomach tissue 1	2	0.000	0	0.245
	Model stomach tissue 2	2		0	
	Model stomach tissue 3	2		2	
	Model stomach tissue 4	2		0	
	Model stomach tissue 5	2		0	

u-MG	Untreated-model stomach tissue 1	3	0.000	1	0.245
	Untreated-model stomach tissue 2	3		1	
	Untreated-model stomach tissue 3	2		0	
	Untreated-model stomach tissue 4	3		0	
	Untreated-model stomach tissue 5	2		0	

TG	Treated stomach tissue 1	1	0.000	0	1.000
	Treated stomach tissue 2	1		0	
	Treated stomach tissue 3	1		0	
	Treated stomach tissue 4	1		0	
	Treated stomach tissue 5	1		0	

^*∗∗*^ *p* < 0.01 of the difference between MG (or TG, u-MG) and BG.

**Table 5:** Information of significant genes (top 20 up and top 20 down).

No.	Gene ID	Gene name	Mean TPM (BG)	Mean TPM (MG)	∣ log_2_Fold change ∣	*p* value	Regulated trend
1	ENSRNOG00000059304	AABR07030529.1	0.1285	4.7350	5.2033	0.048	Up
2	ENSRNOG00000037341	LOC108350059	0.4563	6.5298	3.8388	0.029	Up
3	ENSRNOG00000042680	Krtap14 l	0.2074	2.6476	3.6739	0.024	Up
4	ENSRNOG00000051272	Rn50_20_0464.2	0.1508	1.5527	3.3639	0.022	Up
5	ENSRNOG00000056213	AC130741.1	0.2638	2.1442	3.0228	0.022	Up
6	ENSRNOG00000049770	Cryba4	0.3760	2.9502	2.9720	0.025	Up
7	ENSRNOG00000047915	Igkv8-19	0.5813	4.5120	2.9564	0.044	Up
8	ENSRNOG00000021140	Kcnk4	0.8669	6.1542	2.8276	0.024	Up
9	ENSRNOG00000057458	Oip5	1.1558	5.0659	2.1319	0.021	Up
10	ENSRNOG00000002340	LOC100361079	0.9067	3.8194	2.0746	0.040	Up
11	ENSRNOG00000015878	Pif1	0.9080	3.8173	2.0718	0.027	Up
12	ENSRNOG00000023008	Fam131c	0.8105	3.2547	2.0056	0.031	Up
13	ENSRNOG00000021555	Mis18a	1.4012	5.5511	1.9862	0.035	Up
14	ENSRNOG00000032947	AABR07067749.1	1.0950	4.1869	1.9350	0.019	Up
15	ENSRNOG00000018337	Caly	1.8127	6.5156	1.8458	0.034	Up
16	ENSRNOG00000013292	AC117101.1	2.5861	8.1028	1.6476	0.025	Up
17	ENSRNOG00000029651	Rdh16	0.5834	1.8241	1.6447	0.040	Up
18	ENSRNOG00000020013	Psrc1	2.5036	7.1683	1.5176	0.026	Up
19	ENSRNOG00000028064	Fhad1	0.2188	0.6189	1.5002	0.049	Up
20	ENSRNOG00000055038	AABR07013302.1	3.2159	8.4909	1.4007	0.036	Up
21	ENSRNOG00000049185	LOC100911032	2.8917	0.1098	4.7194	0.029	Down
22	ENSRNOG00000046624	Rn50_2_2278.1	3.0076	0.1423	4.4017	0.040	Down
23	ENSRNOG00000030639	Usp13	6.0753	0.2948	4.3653	0.017	Down
24	ENSRNOG00000048273	Apod	3.6717	0.2367	3.9553	0.023	Down
25	ENSRNOG00000005365	Kcnip1	2.0860	0.1441	3.8553	0.017	Down
26	ENSRNOG00000001873	P2rx6	2.2197	0.1972	3.4925	0.020	Down
27	ENSRNOG00000004575	Il1a	1.4422	0.1352	3.4155	0.048	Down
28	ENSRNOG00000026493	Cdnf	1.3345	0.1339	3.3165	0.022	Down
29	ENSRNOG00000039582	RGD1561161	1.0682	0.1447	2.8840	0.020	Down
30	ENSRNOG00000031716	Ecm2	2.7151	0.3875	2.8088	0.037	Down
31	ENSRNOG00000006645	Ryr3	2.8466	0.4143	2.7804	0.037	Down
32	ENSRNOG00000014509	Sacs	2.2924	0.3669	2.6433	0.026	Down
33	ENSRNOG00000017421	Alpk2	1.0941	0.1758	2.6375	0.033	Down
34	ENSRNOG00000021833	Myrfl	2.0510	0.3380	2.6012	0.039	Down
35	ENSRNOG00000026110	Scml4	0.6388	0.1073	2.5739	0.019	Down
36	ENSRNOG00000031287	Cacna2d3	0.9795	0.1772	2.4663	0.039	Down
37	ENSRNOG00000047545	Adra2a	2.5483	0.6377	1.9986	0.034	Down
38	ENSRNOG00000004812	Sema6d	4.1080	1.2145	1.7581	0.021	Down
39	ENSRNOG00000056359	AABR07035722.1	2.9527	0.9633	1.6160	0.021	Down
40	ENSRNOG00000019751	Cyb5r2	1.9694	0.6469	1.6061	0.020	Down

**Table 6:** Results of relative quantitation of some genes.

Gene name	Sample group	Average Ct value (mean + SEM)	∆Ct (mean + SEM)	∆∆Ct	2^−(∆∆Ct)^	*p* value for ∆Ct

Gapdh	BG	19.9909 ± 0.0818	—	—	—	—
	MG	19.2910 ± 0.4508	—	—	—	—
	TG	19.4393 ± 0.3668	—	—	—	—

Pcsk9	BG	33.4378 ± 0.3312	13.4469 ± 0.3631	0	1	—
	^*∗*^MG	31.4154 ± 1.4045	12.1245 ± 1.0428	−1.3331	2.5194	0.019
	TG	33.5764 ± 0.3318	14.1372 ± 0.1191	0.6796	0.6243	0.200

Arntl	BG	26.6862 ± 0.1382	6.6954 ± 0.1108	0	1	—
	^*∗∗*^MG	27.2743 ± 0.3251	7.9833 ± 0.7463	1.2880	0.4095	0.001
	TG	26.3890 ± 0.2906	6.9498 ± 0.9345	0.2544	0.8383	0.485

^*∗*^represented *p* < 0.05 of the difference between MG (or TG) and BG; ^*∗∗*^represented *p* < 0.01 of the difference between MG (or TG) and BG.

## Data Availability

The data used to support the findings of this study are available from the corresponding author upon request.

## Abbreviations

RT-qPCR: Real-time fluorescence quantitative reverse transcription polymerase chain reaction
FLD: Fuzi Lizhong Decoction
PLS-DA: Partial least-squares discriminant analysis
BG: Blank group
MG: Model group
u-MG: Untreated-model group
TG: Treatment group
TPM: Transcripts per million
KEGG: Kyoto encyclopedia of genes and genomes.

## Supplementary Materials

Supplementary Materials Supplementary Material provides the chromatograph of Fuzi Lizhong Decoction to prove its characterization with multicomponents.
