# Supplementary material for: Transcriptomic Analysis of Fuzi Lizhong Decoction for the Treatment of Stomach Ulcers
Source: Evid Based Complement Alternat Med. 2020 Feb 21;2020:3291853. doi: 10.1155/2020/3291853 (PMC7054793; doi:10.1155/2020/3291853)
Supplement: Supplementary Materials — Supplementary Material provides the chromatograph of Fuzi Lizhong Decoction to prove its characterization with multicomponents. [file 3291853.f1.pdf]

SUPPLEMENTAL DATA

Transcriptomics Analysis of Fuzi Lizhong Decoction for the Treatment of Stomach Ulcers

Yang Xin[1, 2 *], Haijun Wang[3,*], Fengyan Ma[1], Zhe Wang[2], Lei Xu[1]

1 College of Chemistry and Chemical Engineering, Qiqihar University, Qiqihar, 161006, China

2 Heilongjiang Provincial Key Laboratory of Catalytic Synthesis for Fine Chemicals, Qiqihar University, Qiqihar, 161006, China

3 College of Adult and Continuing Education, Qiqihar Medical University, Qiqihar, 161006, China

* **Correspondence:** E–mail address: cc.xinyang@163.com; whjxy0802@163.com

In our previous studies, main several components were qualified and quantified by UPLC/MS technology, FLD in this paper was prepared by the same method with our previous study, the supplementary figures detected by UPLC/MS were to show the figure chromatogram of FLD in this study.

The analytical UPLC-MS system was composed of Acquity Ultra Performance LC and Xevo G2 QTof mass spectrometer with an electrospray source in both positive and negative ion modes (Waters Co., Massachusetts, USA).

The UPLC separation was performed on a CSH C_{18} column (2.1×50 mm, 1.7 μm, Waters Co., USA). The flow rate was set at 0.3 $mL \cdot min^{-1}$. UPLC resolution was optimized with gradient elution as follows: 0～2 min, 2%～20% A; 2～9 min, 20%～100% A; and 9～10 min, 100% A. The column temperature of 30 ℃ could ensure better resolution than 25℃throughout the entire detection process.

The mass spectrometry signal was optimal as follows: capillary (+4.0/-3.5 kV), sampling cone (30 V), extraction cone (4 V), source temperature (100 ℃), desolvation temperature (300℃), cone gas flow (50 $L \cdot h^{-1}$), desolvation gas flow (600 $L \cdot h^{-1}$), scan range (50~2000 Da). Mass accuracy was ensured by using Leucine-enkephalin (Waters Co., USA) as a reference compound.

Fig.S1. basic peak ion chromatograph of Fuzi Lizhong Decoction in positive mode

Fig.S2. basic peak ion chromatograph of Fuzi Lizhong Decoction in negative mode

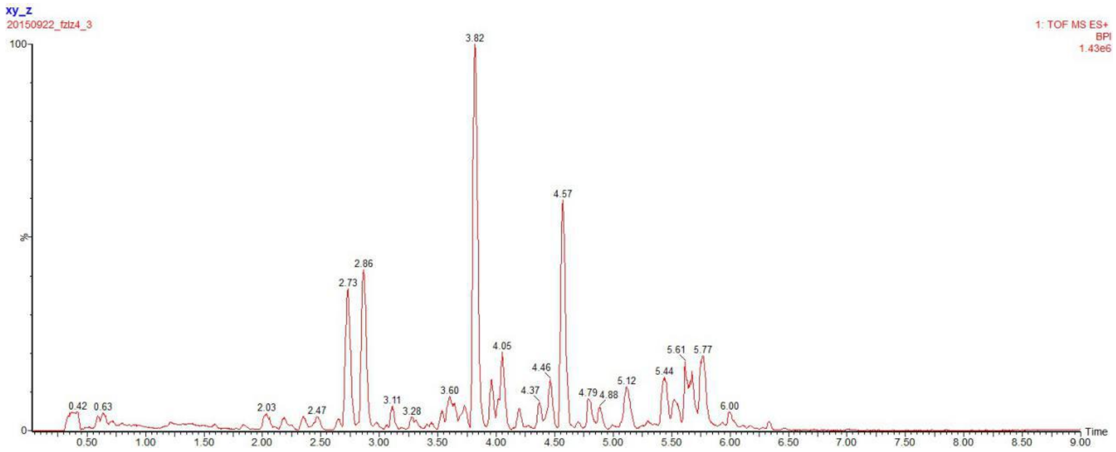

Fig.S1. basic peak ion chromatograph of Fuzi Lizhong Decoction in positive mode

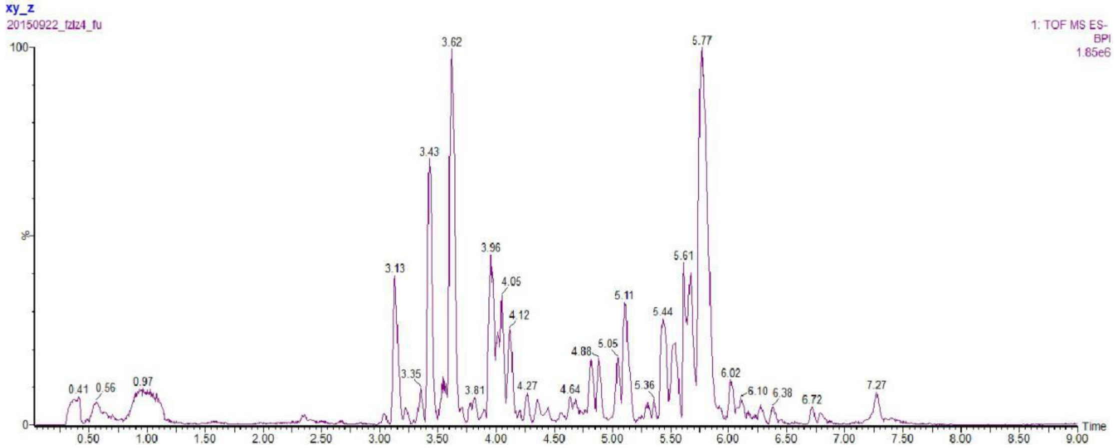

Fig.S2. basic peak ion chromatograph of Fuzi Lizhong Decoction in negative mode
